# The cardiotoxicity of cetuximab as single therapy in Chinese chemotherapy-refractory metastatic colorectal cancer patients

**DOI:** 10.1097/MD.0000000000005946

**Published:** 2017-01-20

**Authors:** Xue-Miao Tang, Hao Chen, Yu Liu, Bin-Lian Huang, Xiu-Quan Zhang, Jian-Mei Yuan, Xia He

**Affiliations:** a Department of Ultrasound and Electrocardiogram; b Department of Clinical Laboratory; c Department of Radiation Oncology; d Department of Nursing, Sun Yat-Sen University Cancer Center; e State Key Laboratory of Oncology in Southern China; f Collaborative Innovation Center for Cancer Medicine, Guangzhou, China.

**Keywords:** cardiac safety, cetuximab, colorectal cancer, targeted therapy, toxicity

## Abstract

The cardiac safety of cetuximab, particularly as single approach, has not been investigated extensively. This trial was designed to evaluate the cardiac safety of cetuximab as salvage monotherapy in Chinese chemotherapy-refractory metastatic colorectal cancer (mCRC) patients.

Cetuximab was administrated at an initial dose of 400 mg/m^2^on day 1 (week 1), followed by a maintenance dose of 250 mg/m^2^ on day 1 of each 7-day cycle. Electrocardiograph (ECG), routine laboratory tests, and troponin I (TNI) Ultra were performed at baseline, during, and after the cetuximab therapy. The incidence of abnormal ECGs, elevated TNI Ultra, cardiac events, and noncardiac events were recorded and analyzed.

TNI Ultra+ was found in 20 patients (32.3%) during the cetuximab therapy.TNI Ultra+ occurred more frequently in patients with more than 3 organs affected and accepted fourth or above lines of chemotherapy. The most frequent abnormal ECG was ST depression in 24 (38.7%) patients. The elevated TNI Ultra and abnormal ECGs could recover after the cetuximab therapy. The most of cardiac adverse events were mild and transient and the noncardiac adverse events were also consistent with the known safety profile for cetuximab.

Cetuximab showed its cardiac safety as a single agent for chemotherapy-refractory mCRC patients. And TNI Ultra and ECG could be sensitive and convenient approaches for the surveillance of adverse events.

## 1 Introduction

Cardiotoxicity is a common complication of many drugs for cancer therapy and pose a serious threaten to the safety and prognosis of patients.^[1]^ The manifestations of cardiotoxicity of anticancer agents include hypotension, ischemia, heart failure, QT prolongation, arrhythmia, and thromboembolism.^[2,3]^ Many traditional cytostatic, especially anthracyclines, cyclophosphamide, fluorouracil, cisplatin, taxanes, vinca alkaloids, and bleomycin are associated with high incidence of cardiotoxicity.^[4]^ With the recent wide application of biological agents, including monoclonal antibodies and tyrosine kinase inhibitors, there are accumulative reports about the cardiotoxicity cause by such drugs such as tratuzumab, lapatinib, bevacizumab, sorafenib, and sunitinib.^[5]^

Cetuximab (Erbitux, Merck KgaA, Darmstadt, Germany) is a chimeric mouse/human antibody against the extracellular domain of epidermal growth factor receptor (EGFR),^[6]^ has effect as single in mCRC patients refractory to irinotecan, oxaliplatin, and fluoropyrimidines, and restores chemosensitivity in irinotecan-refractory mCRC patients.^[7–9]^ The most common side effects of cetuximab including: skin toxicity, diarrhea, hypertension, anorexia, and mucositis/stomatitis are usually reported in the combination with chemotherapy.^[10]^ There are few reports about the cardiac safety of cetuximab, particularly as a single approach.

Our current study was designed to evaluate the cardiac safety of cetuximab by troponin I ultra (TNI Ultra) and 15 leads electrocardiogram (ECG) in a phase II trial of cetuximab as a salvage monotherapy in Chinese chemotherapy-refractory metastatic colorectal cancer (mCRC) patients.

## 2 Methods and patients

### 2.1 Trial design

This study was a phase II trial conducted in Sun Yat-sen university cancer center (Guangzhou, China), designed to evaluate the cardiac safety of cetuximab as salvage monotherapy in Chinese chemotherapy-refractory metastatic colorectal cancer (mCRC) patients. The protocol and all modification have been approved by the Institutional review board and Ethics Committee of our center. The study strictly followed the Declaration of Helsinki and the Good Practice Guidelines. Written informed consent was obtained from every participant.

### 2.2 Eligibility criteria

The eligible criteria of inclusion were: age≥18 years, histologically or cytologically confirmed metastaticadenocarcinoma of the colon or rectum with wild-typeRAS tumor status, measurable or nonmeasurable lesion according to Response Evaluation Criteria In Solid Tumors (RECISTversion 1.1),^[11]^ an Eastern Cooperative Oncology Group performance status (ECOG PS) of ≤2, a life expectancy of more than 3 months, disease progression (clinical or radiological) or intolerance toirinotecan-based and oxaliplatin-based therapy, and had previously received a thymidylate synthase inhibitor (including fluorouracil, capecitabine, raltitrexed) for colorectal cancer.

Exclusion criteria included: previous anti-EGFR therapy, allergy to cetuximab, antitumor therapy within 30 days, central nervous system metastases, clinically significant cardiovascular disease within 1 year before enrollment, active uncontrolled infection, other malignancies in the past 5 years with the exception of adequately treated carcinoma *in situ* of the cervix and squamousor basal cell carcinoma of the skin, inadequate haematological function (absolute neutrophilcount <1.5 × 10^9^/L, platelet count <75 × 10^9^/L, orhaemoglobin <80 g/L), inadequate renal function [creatinine > 1.5 × upper limit of normal (ULN)], and inadequatehepatic function [total bilirubin >1.5 × ULN, or aspartate aminotransferase or alanineaminotransferase >3 × ULN (>5 × ULN if the patient had liver metastases)].

## 3 Treatment

Patients received cetuximabat an initial dose of 400 mg/m^2^ intravenously over 120 minutes on day 1(week 1), followed by a maintenance dose of 250 mg/m^2^ intravenously over 60 minutes on day 1 of each 7-day cycle. The administration of recommended premedication (dexamethasone 10 mg and cimetidine 200 mg) was required before every cycle. The maintenance of cetuximab was continued until disease progression, unacceptable toxicity, planned surgery, serious protocol violation, or patient withdrawal.

## 4 Assessments

An initial 15-lead ECG was performed during the screening and the baseline period (≤7 days prior to registration) using a digital GE-MAC5500 machine and repeated on the day before every cycle.

Blood samples for routine laboratory tests and TNI Ultra were collected 1 hour prior to cetuximab infusion (immediately after the premedication regimen) and at 1 hour after the cetuximab infusion. The concentration of TNI Ultra was determined by a fluorometric enzymeimmunoassay analyzer (Stratus CS, Dade Behring, Miami, FL). The cutoff level of TNI Ultra was 0.78 ng/mL. The elevation of TNI Ultra(TNI Ultra+) was defined as any value exceeding this threshold.

To assess AEs of special interest, some similar AEs were pooled into a composite event category. For example, rash, rash pustular, rash erythematous, dermatitis acneiform, dermatitis exfoliative, rash papular, rash pruritic, rash generalized, rash macular, rash maculopapular, acne, acne pustular, skin desquamation, and dry skin were pooled into the category named acneform rash. The category of infusion reactioncomprised infusion-related reaction, hypersensitivity, anaphylacticreaction, anaphylactic shock, anaphylactoidreaction; dyspnea, pyrexia, and chills during the cetuximab infusion.

Assessments of the efficacy of cetuximab were beyond the scope of this study. Disease response was documented at each evaluation visit (every 6–8 weeks) only for purposes of confirming eligibility for continuing therapy. The radiological approaches (CT, MR, or PET-CT) for response evaluation were decided by the investigators.

## 5 Statistics

The primary endpoint of this study was to determine the cardiac adverse events (AEs) in the study population according to the NCI-CTCAE version 4.02.

The secondary endpoint was to evaluate the common noncardiac AEs.

All statistical tests were 2-sided, and significance was assumed at *P*<0.05. Results are presented as mean ± standard deviation, unless otherwise specified. The *t*-test was used for normally distributed continuous variables, and either the χ^2^ or Fisher's exact test was used for categorical variables.

Data were recorded and analyzed with SPSS (version 23), GraphPad Prism (version 6.0).

## 6 Results

**Figure 1:**
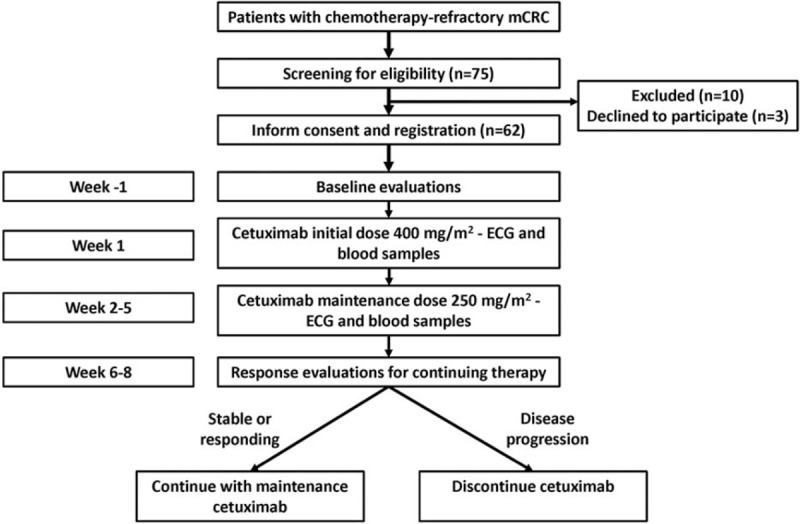
Study diagram.

Between September 1 2013 and January 1 2015, 75 patients were screened for this study and 62 of them (40 males and 22 females) were enrolled finally (10 patients were excluded and 3 patients declined to participate) (Fig. 1). Baseline demographic and clinical characteristics were summarized in Table 1. The patients received a median of 28 cycles (range 18–48 cycles) of cetuximab. The median follow-up duration after cetuximab discontinuation was 8 months (range, 3–17 months).

**Table 1:**
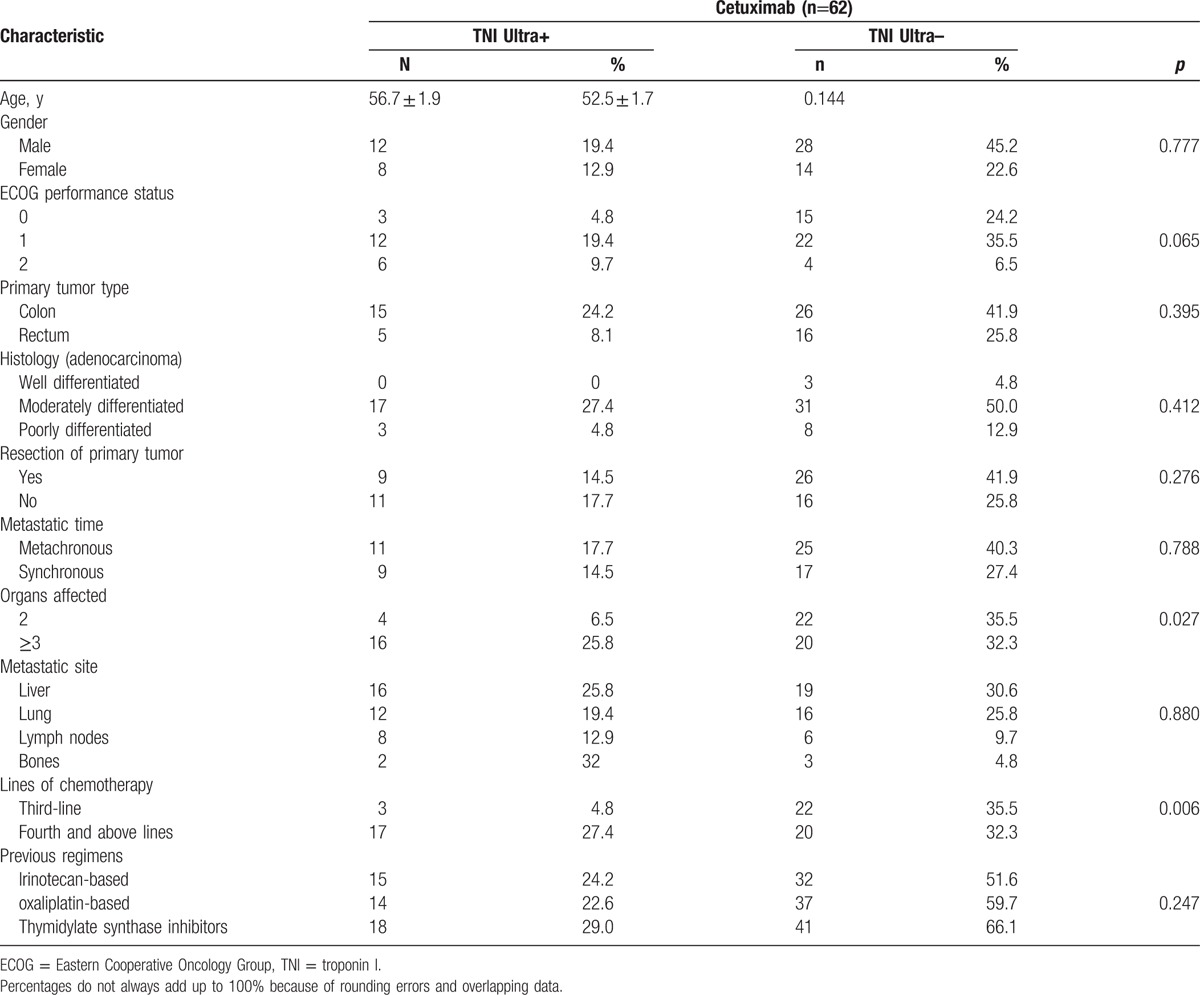
Clinical characteristics of patients with TNI Ultra+ or TNI Ultra.

### 6.1 TNI Ultra

**Figure 2:**
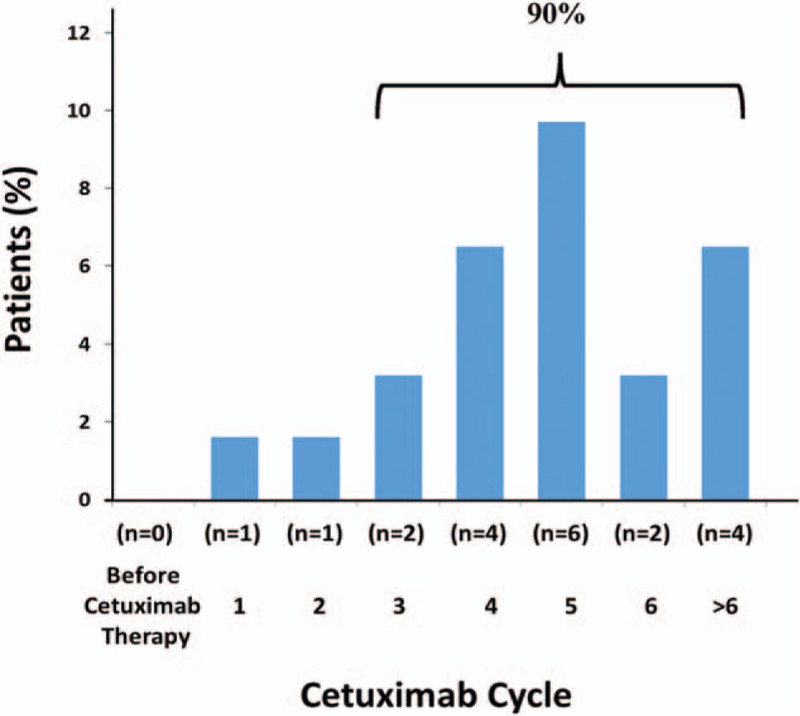
Time of the first detection of elevated TNI Ultra. TNI = troponin I.

All patients have normal TNI Ultra at baseline. TNI Ultra+ was found in 20 patients (32.3%, mean value: 0.84 ± 0.27 ng/mL) during the cetuximab therapy. The clinical characteristics of patients with TNI Ultra+ or TNI Ultra– was summarized in Table 1. TNI Ultra+ occurred more frequently in patients with more than 3 organs affected and accepted fourth or above lines of chemotherapy. Interestingly, the first TNI Ultra+ was observed, in most cases (18/20, 90%), after the 3rd cetuximab cycle (Fig. 2). And all of elevated TNI Ultra levels recovered at 3 months after cetuximab therapy.

### 6.2 ECG

**Figure 3:**
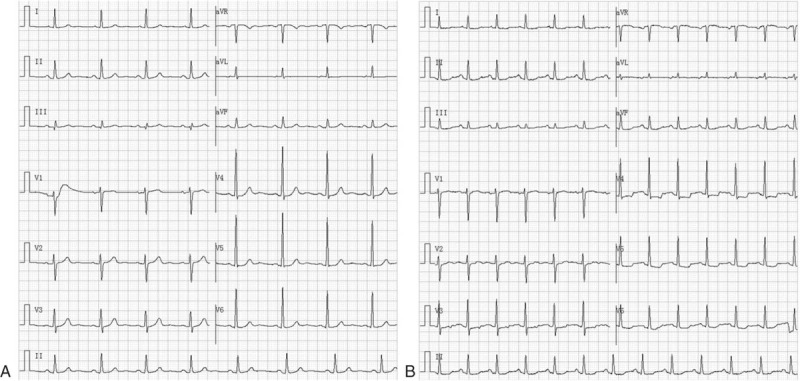
Typical ST depression in ECG: (A) baseline; (B) during the cetuximab therapy. ECG = electrocardiograph.

There were 4 cases of atrial arrhythmia, 2 cases of reverse T wave, and 1 case of prolonged QRS interval in baseline ECG. During the cetuximab treatment, the most frequent abnormal ECG was ST depression (whole leads or chest leads), which can be observed in 24 (38.7%) patients (Fig. 3). Other frequent abnormal ECG included atrial arrhythmia, prolonged QRS interval and reverse T wave. Severe abnormalities such as prolonged QT interval haven’t been observed. At 3 months after cetuximab treatment, there were still 3 cases of atrial arrhythmia and 1 case of reverse T wave. Other abnormal ECGs have all recovered (Table 2).

**Table 2:**
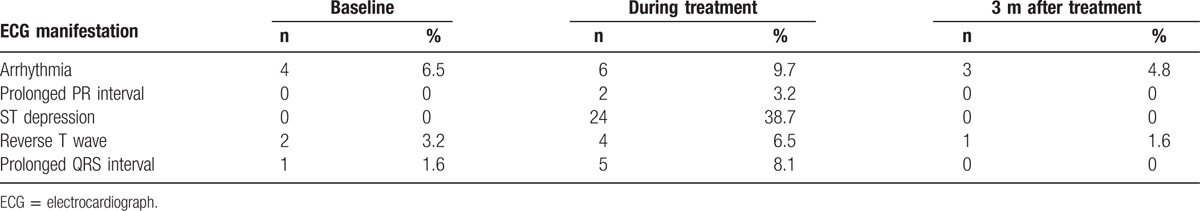
Summary of ECG abnormalities.

### 6.3 Cardiac adverse events

Overall, there were 24 cardiac adverse events occurred during the cetuximab treatment (Table 3). There were not severe events such as acute coronary syndrome, heart failure, and cardiac death be observed. Except for 1 case of grade 3 chest pain need intervention (oxygen inhalation, electrocardiogram monitoring, and administration of nitroglycerin), all events were mild and could recover without interventions.

**Table 3:**
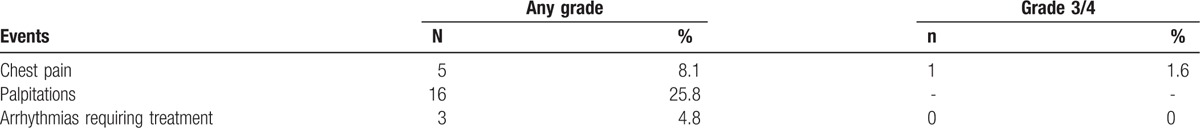
Major cardiac adverse events in the overall study population.

### 6.4 Noncardiac adverse events

Acneform rash was still the most frequent noncardiac adverse events during the cetuximab treatment. There were 51 cases (82.3%) of acneform rash in the overall study population and 24 of them were grade 3/4. Other frequent events included fatigue, diarrhea, stomatitis, and infusion reaction. The hematologic toxicities were rare and mild (Table 4).

**Table 4:**
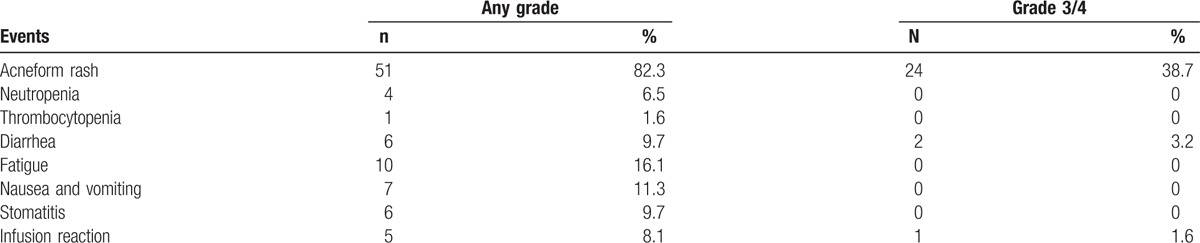
Major noncardiac adverse events in the overall study population.

## 7 Discussion

The use of cetuximab in mCRC patients with wild-type RAS gene has significantly improved the prognosis.^[7–9,12,13]^ However, the cardiac safety profile of cetuximabin mCRC patients, particularly as a single approach for salvage therapy, has not been studied previously. The mCRC patients usually received multiple lines of chemotherapy. Although the cardiac toxicities are not common in main agents used for mCRC, such as oxaliplatin, irinotecan, capecitabine, if the accumulative nonspecific toxicities of previous chemotherapy will increase the risk of cardiac adverse events of cetuximab is unclear.

For cancer patients with high-dose chemotherapy, troponin I (TNI) is a well-established early marker of myocardial injury, with high sensibility and specificity.^[14–16]^ With the use of ultra-sensitive troponin I (TNI-Ultra), the sensibility and specificity in diagnosis of myocardial injury have been further improved.^[17,18]^ The elevation of TNI-Ultra has been shown to predict the presence and outcome of adverse cardiac events. But the significance of TNI-Ultra in cetuximab-treatment mCRC patients has not been investigated.

In our study, all patients have normal TNI Ultra at baseline. TNI Ultra+ was found in 20 patients (32.3%, mean value: 0.98 ± 0.27 ng/mL) during the cetuximab therapy. The analysis of association of TNI Ultra+ with clinicopathological characteristic revealed that TNI Ultra+ occurred more frequently in patients with more than 3 organs affected and accepted fourth or above lines of chemotherapy. It suggested that relative heavier tumor burden and excessive chemotherapy might contribute to the higher incidence of TNI Ultra+. We also found that most (18/20, 90%) of first TNI Ultra+ was observed after the 3rd cetuximab cycle. And all of elevated TNI Ultra levels recovered at 3 months after cetuximab therapy. We assumed that the effect of cetuximab on TNI Ultra might be dose-dependent and accumulated.

In the evaluation of ECG during cetuximab therapy, the most frequent abnormal ECG was ST depression (whole leads or chest leads), which can be observed in 24 (38.7%) patients. ST depression usually indicates the presence of myocardial ischemia or injury.^[19,20]^ The incidence of abnormal ST is consistent with the elevation of TNI Ultra in the study population and they were both transient and reversible. Prolonged QT interval is one of the severe abnormalities of ECG which may lead to malignant ventricular tachyarrhythmias such as torsades de pointes, which can quickly progress to ventricular fibrillation and sudden death.^[21,22]^ Our results suggested that cetuximab can besafely administered as a single agent without risk of prolonged QT interval, which is consistent with the study of the cetuximab in other advanced solid tumors by Deeken et al.^[23]^

The most common cardiac events of cetuximab in our study included palpitations (25.8%), chest pain (8.1%) and arrhythmias requiring treatment (4.8%). Most of the events were mild and transient. Palpitations and chest pain usually occurred with rapid infusion of cetuximab and could be relieved with adjustment of infusion speed. The noncardiac adverse events in our study were also consistent with the known safety profile for cetuximab. There were not new safety concerns for cetuximab revealed by clinical and laboratory data.

Trastuzumab, a humanized monoclonal antibody targeting the HER2 receptor used in treatment for metastatic breast cancer, is a typical biological agent associated with cardiac toxicities such as decreased left ventricular ejection fraction, particular in combination with anthracycline.^[5,24]^ While lapatinib, a tyrosine kinase inhibitoralso targeting HER2, has been associated with QTprolongation.^[3]^ As a chimeric mouse/human antibody, cetuximab showed transient and reversible effects on TNI ultra and ECG in our study. Because of the lack of enough evidence, we hypothesized that the antibody-dependent cell-mediated cytotoxicity (ADCC) might partially be the underlying mechanism.

Collectively, cetuximab showed its cardiac safety as a single agent for chemotherapy-refractory mCRC patients in our study. But monitoring for cardiac adverse events is still necessary, and TNI Ultra and ECG could be sensitive and convenient approaches for the surveillance.
